# Trends in Dietary Intake of Total Fat and Fatty Acids Among Korean Adolescents from 2007 to 2017

**DOI:** 10.3390/nu11123073

**Published:** 2019-12-16

**Authors:** SuJin Song, Jae Eun Shim

**Affiliations:** 1 Department of Food and Nutrition, Hannam University, Daejeon 34054, Korea; sjsong@hnu.kr; 2 Department of Food and Nutrition, Daejeon University, Daejeon 34520, Korea

**Keywords:** total fat, saturated fatty acids, polyunsaturated fatty acids, adolescents, Korea

## Abstract

We analyzed the trends in dietary intake of total fat and fatty acids among Korean adolescents during 2007–2017. A total of 6406 adolescents from the 2007–2017 Korea National Health and Examination Surveys were selected. Total fat and fatty acids intakes were calculated based on single 24-hour recall data and presented as grams (g) and percentage of energy intake (% kcal) across the survey period. Linear trends in intake across the survey period were compared using the multiple regression model. Total fat intake increased during the 11-year period from 54.3 g (21.7% kcal) to 61.8 g (25.2% kcal). Saturated fatty acids (SFA) and monounsaturated fatty acids (MUFA) intakes changed from 17.8 g (7.1% kcal) and 17.2 g (6.8% kcal) to 20.6 g (8.4% kcal) and 20.7 g (8.4% kcal) over time, respectively. For polyunsaturated fatty acids (PUFA), n-3 fatty acid intake did not change during the survey period. The proportions of individuals who had total fat and SFA above the recommendations increased across the survey period: 13.7% to 27.5% for total fat and 36.0% to 49.7% for SFA. Among Korean adolescents, dietary fat intake increased over time and the increases in SFA and MUFA intake were prominent. Monitoring dietary fat intake is helpful to suggest dietary guidelines and health policies.

## 1. Introduction

Cardiovascular diseases are highly prevalent and major causes of mortality worldwide [1]. Accordingly, for reducing cardiovascular disease risks, dietary guidelines by different organizations suggest limiting total fat intake within 20–35% of energy and replacing saturated fatty acids (SFA) intake with polyunsaturated fatty acids (PUFA) intake [2,3,4,5]. Recently, early development of cardiovascular diseases risk factors in childhood and adolescence have been reported [6,7], and the presence of cardiovascular risk factors in adolescence might extend to the risk of chronic diseases in adulthood [8,9]. Therefore, adequate intake of dietary fat, including various types of fatty acids, is of great importance for the health status in adolescence as well as in later adult life.

Assessing dietary intake of total fat and fatty acids among adolescents has revealed that adolescents in Asian countries still showed lower intakes of total fat and SFA than in Western countries [10,11,12,13,14]. In terms of changes in dietary fat intake over time, Chinese adolescents showed a rapid increase in dietary fat intake [11] whereas European or U.S. adolescents showed no significant changes in total fat or SFA intake. [15,16,17,18]. In addition, German and Australian studies observed a decreasing trend in PUFA intake among adolescents [18,19].

In Korea, along with development of the food industry and changes of lifestyle of individuals, nutrition transition has been observed, which is characterized by a higher consumption of animal foods, sugars, and processed foods [20]. Based on the data from the Korea health statistics, fat consumption has increased, whereas carbohydrate intake has decreased, since 1998 in the Korean population [21]. This report also showed that chronic health conditions, such as obesity, hyperlipidemia, and diabetes, are highly prevalent in Korea [21]. A recent study monitoring the trend in total fat and fatty acids intake in Korean adults showed that dietary fat intake increased over time, and an increase in SFA and monounsaturated fatty acids (MUFA) intake was prominent [22]. Furthermore, another Korean study found that the proportions of individuals who consumed more than the recommended level for total fat and SFA were relatively higher in younger adults than in older adults [23]. A recent systematic review reported that European adolescents did not meet the dietary guidelines for fat intake [12]: total fat and SFA intake exceeded the recommended level, but PUFA intake was below the recommendation.

There has been no focus on investigating changes in total fat and fatty acids intake among Korean adolescents. The prevalence of obesity among Korean adolescents has been increasing from 8.3% in 2006 to 12.9% in 2015 [24]. Consequently, continuous increases in prevalence of chronic diseases, such as cardiovascular disease, type 2 diabetes, and dyslipidemia in Korea could be predicted. To identify targets for nutrition intervention to prevent chronic disease, it is necessary to evaluate dietary fat intake over time in Korean adolescents. Monitoring changes in dietary patterns of the population can be helpful for designing effective dietary guidelines and health policies. Therefore, we analyzed the trends in dietary intake of total fat and fatty acids among Korean adolescents during 2007–2017 using the national survey data.

## 2. Materials and Methods

### 2.1. Data and Study Subjects

We used data from the fourth (2007–2009), fifth (2010–2012), sixth (2013–2015), and seventh (2016–2017) Korea National Health and Examination Surveys (KNHANES) to calculate trends in total fat and fatty acids intake in Korean adolescents. The KNHANES is a cross-sectional and nationally representative survey which is intended to assess and monitor health and nutritional status of Koreans. It is conducted by the Korea Centers for Disease Control and Prevention every year and consists of a health interview, physical examination, laboratory test, and nutrition survey. Detailed descriptions of the KNHANES have been published elsewhere [25].

The KNHANES selects survey samples of the civilian noninstitutionalized Korean population using a stratified, multistage probability sampling design. Among the eligible samples who were aged 12–18 years and had dietary data from the 4th, 5th, 6th, and 7th KNHANES (*n* = 6620), we excluded individuals who had no information on household income (*n* = 82) and reported an implausible energy intake (<1st or >99th percentile of energy intake by gender, *n* = 132). A total of 6406 adolescents (3361 boys and 3045 girls) were included in the final data analyses. This study was conducted in accordance with the Declaration of Helsinki and was approved by the Korean Centers for Disease Control and Prevention Institutional Review Board. Written informed consent was obtained from each subject.

### 2.2. Dietary Assessment

Dietary data used in this study were obtained from a single 24-hour dietary recall and were collected by trained dietitians at each subject’s home, about one week after the completion of the health interview and examination. Energy and macronutrient intake were calculated based on the Food Composition Table published by the Korean Rural Development Administration [26,27,28]. Macronutrient intake was presented as proportions of total energy intake.

A database on fatty acids contents of common Korean foods was used to calculate intake of SFA, MUFA, PUFA, n-3 fatty acids (n-3 FA), and n-6 fatty acids (n-6 FA) for each subject. The database of fatty acids was developed in 2013, and the KNHANES has released the data on fatty acids intake of the survey participants since the 6th KNHANES [29]. For the 4th and 5th KNHANES, which had no data on fatty acids intake, the fatty acid intake was calculated using the fatty acid contents per 100 g of foods that appeared in the 6th KNHANES dataset. For 997 food items that did not appear in the 6th KNHANES, the fatty acid contents were replaced with calculated or imputed values using the fatty acid contents of similar Korean foods (922 items, 92%) or of foods in the U.S. Department of Agriculture fatty acid database (39 items, 4%). About 4% (36 items) of food items which had very low fat contents and no reference data were considered as the content of fat was zero. For foods that was consisted of several food items (e.g., sandwich, pizza, hamburger, or fried rice with shrimp), the fatty acid contents of the main food source for total fat were applied. Total fat and fatty acids intake were presented as grams (g) and percentage of energy intake from each fatty acid (% kcal). To evaluate total fat and SFA intake, the acceptable macronutrient distribution range (AMDR) from the 2015 Dietary Reference Intake for Koreans was used: the AMDR for total fat was 15–30% and for SFA is <8% in adolescents [30].

### 2.3. Sociodemographic Variables

Information on sociodemographic characteristics (e.g., gender, age, region, and household income) were obtained during the health interview using a questionnaire. Region was divided into urban or rural areas based on administrative districts. Household income was categorized as lowest, medium-low, medium-high, or highest.

### 2.4. Statistical Analyses

We did all statistical analyses using the Statistical Analysis Systems (SAS) software package, version 9.4 (SAS Institute, Cary, NC, USA). All analyses accounted for the complex sampling-design effect and used appropriate sampling weights to produce estimates of all Korean adolescents from the representative survey sample. Intake of total fat and fatty acids was presented as means and standard errors, and sub-analysis was done by gender, age, region, and household income. Linear trends in intake of total fat and fatty acids across the survey periods were compared using multiple regression after adjustment for gender, age, region, and household income, where applicable. Proportions of subjects who had more than the dietary guidelines for total fat and SFA were presented as percentages and standard errors across survey periods. All *p* < 0.05 were considered statistically significant.

## 3. Results

### 3.1. Characteristics of the Study Subjects

Table 1 presents the characteristics of the study subjects by the survey period. A total of 6406 Korean adolescents was included in this study; of them, 54% were boys. About 60% of the subjects were at high-school level (15–18 years) and 84% lived in urban areas. Distribution of subjects by gender, region, and household income did not change according to the survey period. The proportion of high-school adolescents was significantly higher in the 2016–2017 survey than in the 2007–2009 survey. In this study population, the percentage of energy from carbohydrate intake decreased from 64% kcal in 2007–2009 to 60% kcal in 2016–2017, whereas fat intake increased from 22% kcal in 2007–2009 to 26% kcal in 2016–2017 (*p* < 0.0001).

### 3.2. Trends in Total Fat Intake

Table 2 shows trends in dietary intake of total fat during 2007–2017. The Korean adolescents showed a significant increase in total fat intake as well as energy from fat from 2007 through 2017: 54.3 g to 61.8 g in total fat intake and 21.7% kcal to 25.2% kcal in energy from fat. In all sub-groups by gender, age, and region, the total fat intake (in grams and % kcal) significantly increased across the survey period. Higher income groups showed a more prominent increase in total fat intake assessed as amount and proportion of energy than did those in lower income groups.

### 3.3. Trends in Fatty Acids Intake

Trends in dietary intake of SFA, MUFA, and PUFA are presented in Table 3, Table 4 and Table 5, respectively. SFA intake increased from 17.8 g to 20.6 g from 2007 through 2017, as did the proportion of energy from SFA, from 7.1% kcal to 8.4% kcal (*p* < 0.0001). By gender, age, and region, all sub-groups showed significant increases of SFA intake across the survey period. Adolescents with lower household income did not show a significant increase in SFA intake, whereas those with higher household income did. MUFA intake was 17.2 g (6.8% kcal) in 2007–2009 and 20.7 g (8.4% kcal) in 2016–2017 (*p* < 0.0001) among total study subjects. Energy intake from MUFA also significantly increased in all gender, age, region, and household income groups during 2007–2017. The trend in PUFA intake was different from the trend for total fat, SFA, and MUFA. The n-3 FA intake did not show any changes during the survey period, except in the group of 12–14 years. In terms of PUFA intake, an increase of n-6 FA intake was more prominent than that of n-3 FA intake.

### 3.4. Trends in Prevalence of Excessive Intake of Total Fat and SFA

**Figure 1:**
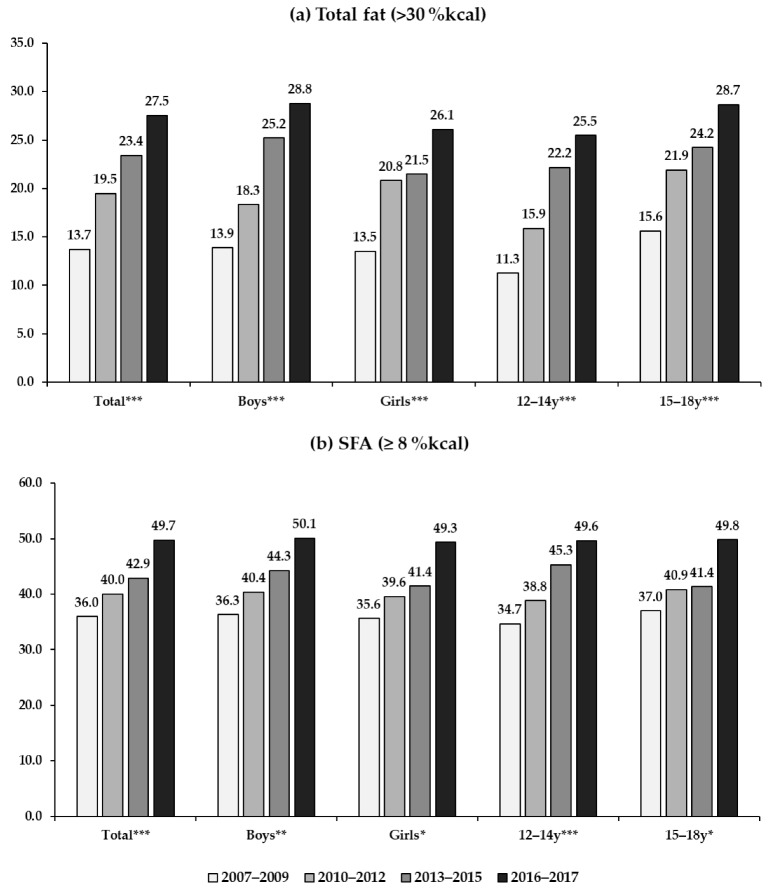
Trends in proportions of subjects who had more than the dietary guidelines for total fat and saturated fatty acids (SFA) among Korean adolescents during 2007–2017: (**a**) total fat; (**b**) SFA. (**a**) The statistical analysis accounted for the complex sampling-design effect and used appropriate sample weights. (**b**) *p* trends were obtained from the chi-square test to examine differences in proportions across the survey period. * < 0.01, ** < 0.001, *** < 0.0001.

Figure 1 shows the time trend in proportions of individuals who had more than the dietary guidelines for total fat and SFA during 2007–2017. The Korean adolescents who consumed more than 30% of energy from total fat significantly increased, from 13.7% to 27.5% during 2007–2017. All gender and age groups showed significant increases (*p* < 0.0001). Boys and younger adolescents showed steeper increases compared to girls and older adolescents, respectively. The proportion of individuals who had more than 8% of energy from SFA was 36.0% in 2007–2009 and 49.7% in 2016–2017. In 2016–2017, about half the study population consumed SFA above the recommended level in all gender and age groups.

## 4. Discussion

In this study, we found substantial increases in dietary intake of total fat, SFA, and MUFA during 2007–2017 among Korean adolescents, although the trend in PUFA intake was different. Increasing trends in total fat, SFA, and MUFA intake were seen in all sub-groups classified by gender, age, and region, whereas changes of PUFA intake in some sub-groups were not significant. Particularly, n-3 FA intake did not show any changes during the survey years except for the 12–14 years age group. In terms of evaluation of total fat and SFA intake based on the AMDR level, proportions of individuals who had total fat and SFA above the recommended level gradually increased across the survey period: from 13.7% to 27.5% for total fat and from 36.0% to 49.7% for SFA.

This study showed prominent increases in intake of total fat and SFA in terms of absolute amount as well as energy contribution in Korean adolescents. About 7.5 g (3.5% kcal) of increase in total fat was observed during an 11-year period; this increase was mainly attributed to increases of SFA (2.8 g; 1.3% kcal) and MUFA (3.5 g; 1.6% kcal) intake. Recent survey data showed that the proportion of high school students with relatively high levels of fat intake was high, and the trend of increasing fat intake was reflected by this characteristic. However, the main findings of the increase in the level of fat intake of adolescents remain unchanged since both middle and high school students showed a clear increase in intake. Chinese children and adolescents aged 7–17 years also showed significant increases in total fat intake, from 54.8 g in 1991 to 66.0 g in 2009, and this trend led to an increase in the proportion of individuals having ≥30% kcal from fat: 20.1% in 1991 to 49.4% in 2009 [11]. In addition, increasing trends in total fat, SFA, and MUFA intake were consistently found in adults from Asian countries [22,31,32], which might be explained by the nutrition transition that occurred in Asian countries. However, in a sample of German adolescents aged 13–18 years, absolute intake of total fat, SFA, and MUFA did not significantly change between 2000 and 2010, whereas PUFA intake slightly decreased by about 0.2% kcal per year [18]. The US children and adolescents showed no changes in % kcal from total fat and SFA between 1999 to 2000 and 2009 to 2010 [16], and the UK adolescents aged 11–18 years showed that total fat and SFA intake (g and % kcal) slightly decreased [15].

Surprisingly, our analysis found that n-3 FA intake did not change during the survey period among Korean adolescents, even though total fat intake markedly increased. In our data, PUFA intake slightly increased by 0.8 g (0.4% kcal) between 2007 and 2017 in a total study sample, but significant upward trends in PUFA were only observed in the boys, 12–14 years, urban area, and medium-high income groups. Among German adolescents, absolute intake of PUFA did not change and the percentage of energy from PUFA slightly decreased between 2000 and 2010 [18]. Australian adolescents aged 12–18 years also showed a decline of PUFA and n-3 FA intake from 1995 to 2012 [19]. In line with these results, recent reports described that adolescents from developed countries did not meet the recommendations for PUFA or n-3 FA intake [12,33,34]. A previous study of Korean adults showed that PUFA intake increased steadily but was more prominent in n-6 FA intake than in n-3 FA intake [22]. Taken together, both the present findings and the findings from the Korean adults demonstrate that total fat intake substantially increased in Korea, which was mainly observed in increases of SFA and MUFA intake, not PUFA intake, including n-3 FA.

The Dietary Reference Intake for Koreans suggests the AMDR as 15–30% kcal of total fat and <8% kcal of SFA for adolescents [30]. Using this recommendation, we evaluated total fat and SFA intake among Korean adolescents and found that proportions of individuals who were above the recommendation increased across survey years. Our data in 2016–2017 described that about 28% and 50% of adolescents did not meet the recommendation for total fat and SFA, respectively. In the Korean adult population, young adults aged 19–29 years also showed a relatively higher proportion who exceeded the recommendation level for total fat and SFA than did middle-aged or older adults [23]. The Korean AMDR has a lower recommended level for total fat and SFA since a Korean diet shows a high carbohydrate but a low fat consumption. Compared to this, current guidelines in Western countries typically suggest 20–35% kcal for total fat and <10% kcal for SFA [35]. Adolescents from developed countries showed much higher proportions exceeding the recommendation of total fat and SFA even though they had a higher recommended level for total fat and SFA [12,18,33]. Considering our findings, future dietary guidelines should focus on the improvement of fat intake by replacing SFA intake with PUFA, especially n-3 FA intake within the recommended range for total fat intake. In addition, nutrition education that emphasizes food practices for reducing SFA and increasing PUFA intake are needed in this population.

Although estimations on dietary fat intake from each study were obtained from different dietary assessment methods and food composition tables, as well as different time frame, Asian adolescents still showed an intake of total fat and SFA lower than that of adolescents in Western countries [11,12,15,16,18]. However, Asian adolescents showed rapid increases in dietary fat intake, particularly SFA and MUFA intake. In terms of food sources of fat intake, intake of total fat, SFA, and MUFA in Korean adolescents was mainly from pork, soybean oil, instant noodles, beef, and eggs [10]. Similarly, the food sources of PUFA and n-3 intake were reported as soybean oil, pork, mayonnaise, tofu, and eggs [10]. Future studies focusing on changes of food sources of dietary fat in the Korean population will be needed. Furthermore, associations of the type and sources of dietary fat intake with the development of non-communicable diseases based on a cohort design study should be investigated to set up specific dietary recommendations for fat intake.

This study has several limitations. Dietary intake of total fat and fatty acids were assessed based on single 24-hour recall data. However, this study focused on the population-level consumption of dietary fats. Because the fatty acid composition table has been available since the 2013 KNHANES, dietary fatty acids intake of participants in the 2007–2012 KNHANES were calculated using the contents of fatty acids of foods listed in the 2013 KNHANES, which might be different from the actual contents of fatty acids in foods that appeared in the 2007–2012 KNHANES. The assessment of fat intake levels with AMDR has limitations. If the total energy intake level is very low, even if the fat intake ratio is high, the absolute fat intake level may be low, and the intake of essential fatty acids may be insufficient. However, in assessing the average intake of a population, it might not distort the overall intake patterns. Despite these limitations, to our knowledge, this was the first study to evaluate trends in dietary intake of total fat and fatty acids during an 11-year profile among Korean adolescents using a nationally representative sample. Since the prevalence of overweight and obesity among Korean adolescents has been increasing, dietary assessment and monitoring of total fat and fatty acids intake is significant for providing effective dietary guidelines and health policies.

In conclusion, our investigation provided information on temporal trends of dietary fat intake based on quantitative estimates of the national-level consumption by gender, age, region, and household income among Korean adolescents. Our results demonstrated that dietary intake of total fat, including SFA, and MUFA, markedly increased, whereas PUFA intake, especially n-3 FA, did not show any changes during the 11-year period. Because adolescence is the critical period for nutritional health status through a person’s lifetime, dietary evaluations are important to identify nutritional health problems and then provide appropriate nutrition interventions. Future research focusing on food-intake patterns in terms of types or sources of dietary fats are needed to suggest food-based dietary guidelines for this population. Furthermore, associations of fatty acids intake with health outcomes, such as chronic diseases, should be examined.

## Figures and Tables

**Table 1:** Characteristics of the study subjects by the survey period ^1^.

Characteristics	Total (n = 6406)		2007–2009 (n = 1931)		2010–2012 (n = 1883)		2013–2015 (n = 1633)		2016–2017 (n = 959)		*p*-Value ^2^
	% or Mean	SE	% or Mean	SE	% or Mean	SE	% or Mean	SE	% or Mean	SE	
**Gender**											
Boys	53.5	0.3	53.8	1.1	54.8	1.2	51.8	1.1	53.4	1.2	0.4104
Girls	46.5	0.3	46.2	1.0	45.2	1.1	48.2	1.1	46.6	1.2	
**Age**											
12–14 years	40.1	0.3	43.8	0.9	40.5	0.9	39.1	0.9	35.6	0.9	0.0013
15–18 years	59.9	0.4	56.2	1.2	59.5	1.4	60.9	1.2	64.4	1.5	
**Region**											
Urban	83.6	0.5	82.4	1.7	82.0	1.9	84.1	1.8	87.4	1.9	0.3855
Rural	16.4	0.3	17.6	1.2	18.0	1.4	15.9	1.1	12.6	0.9	
**Household income**											
Lowest	13.2	0.2	12.9	0.6	14.6	0.8	12.9	0.6	11.8	0.7	0.1890
Medium-low	26.6	0.3	23.4	0.8	29.1	1.2	27.3	0.9	25.7	0.9	
Medium-high	30.5	0.3	32.3	1.0	27.7	1.0	31.6	1.0	31.1	1.0	
Highest	29.7	0.3	31.4	1.1	28.6	1.0	28.2	1.1	31.4	1.2	
**Energy and macronutrient intake**											
Total energy (kcal)	2129	12.7	1970	20.3	2215	24.9	2189	24.3	2121	32.3	<0.0001
Carbohydrate (% kcal)	62.0	0.2	64.0	0.3	62.4	0.3	61.2	0.3	59.9	0.4	<0.0001
Fat (% kcal)	23.6	0.1	21.9	0.2	23.3	0.3	24.3	0.2	25.5	0.3	<0.0001
Protein (% kcal)	14.4	0.1	14.1	0.1	14.4	0.1	14.5	0.1	14.6	0.2	0.0079

SE, standard error. ^1^ The statistical analysis accounted for the complex sampling design effect and used appropriate sample weights. ^2^ *p*-values were obtained from the chi-square test to examine differences in distribution of variables across the survey period.

**Table 2:** Trends in total fat intake among Korean adolescents during 2007–2017 ^1^.

Characteristics	Total Fat (g) ^2^									Total Fat (% kcal) ^3^								
	2007–2009		2010–2012		2013–2015		2016–2017		*p*-Trend	2007–2009		2010–2012		2013–2015		2016–2017		*p*-Trend
	Mean	SE	Mean	SE	Mean	SE	Mean	SE		Mean	SE	Mean	SE	Mean	SE	Mean	SE	
**Total**	54.3	0.7	56.2	0.8	57.3	0.7	61.8	1.0	<0.0001	21.7	0.3	23.1	0.3	24.1	0.3	25.2	0.4	<0.0001
**Gender**																		
Boys	61.4	1.0	63.6	1.1	64.8	1.0	69.9	1.5	<0.0001	21.9	0.3	23.2	0.4	24.4	0.4	25.6	0.5	<0.0001
Girls	46.4	0.8	48.1	1.0	49.1	0.8	52.9	1.1	<0.0001	21.5	0.4	23.0	0.4	23.8	0.4	24.8	0.5	<0.0001
**Age**																		
12–14 years	52.0	0.7	53.7	0.9	56.0	0.9	59.7	1.2	<0.0001	21.0	0.3	22.2	0.4	23.9	0.4	24.6	0.5	<0.0001
15–18 years	56.2	1.0	58.4	1.1	58.7	0.9	63.7	1.3	<0.0001	22.3	0.4	23.8	0.4	24.4	0.4	25.8	0.5	<0.0001
**Region**																		
Urban	54.7	0.7	56.3	0.8	57.6	0.7	61.7	1.0	<0.0001	21.9	0.3	23.1	0.3	24.2	0.3	25.3	0.4	<0.0001
Rural	52.2	1.2	55.1	1.5	56.0	1.2	62.4	2.7	0.0002	21.3	0.5	23.2	0.6	24.2	0.6	25.4	1.1	<0.0001
**Household income**																		
Lowest	50.6	2.1	53.7	1.7	53.3	1.7	57.6	2.0	0.0386	21.6	0.6	23.5	0.8	23.3	0.8	24.8	0.9	0.0059
Medium-low	54.9	1.2	54.8	1.4	53.0	1.2	59.6	2.3	0.1784	22.4	0.5	22.9	0.5	23.2	0.5	24.9	0.8	0.0066
Medium-high	53.7	1.2	56.7	1.4	59.1	1.1	63.8	1.5	<0.0001	21.2	0.4	22.9	0.6	24.5	0.5	25.7	0.6	<0.0001
Highest	56.2	1.1	57.8	1.6	61.3	1.3	63.3	1.8	<0.0001	22.1	0.4	23.5	0.6	25.2	0.5	25.4	0.7	<0.0001

SE, standard error. ^1^ The statistical analysis accounted for the complex sampling design effect and used appropriate sample weights. ^2^ Means (SE) and *p* trends were obtained from the multiple regression after adjustment for gender, age, region, household income, and total energy intake, where applicable. ^3^ Means (SE) and *p* trends were obtained from the multiple regression after adjustment for gender, age, region, and household income, where applicable.

**Table 3:** Trends in saturated fatty acids (SFA) intake among Korean adolescents during 2007–2017 ^1^.

Characteristics	SFA (g) ^2^									SFA (% kcal) ^3^								
	2007–2009		2010–2012		2013–2015		2016–2017		*p*-Trend	2007–2009		2010–2012		2013–2015		2016–2017		*p*-Trend
	Mean	SE	Mean	SE	Mean	SE	Mean	SE		Mean	SE	Mean	SE	Mean	SE	Mean	SE	
**Total**	17.8	0.3	18.4	0.3	18.4	0.3	20.6	0.4	<0.0001	7.1	0.1	7.6	0.1	7.8	0.1	8.4	0.2	<0.0001
**Gender**																		
Boys	20.4	0.5	21.3	0.5	20.8	0.5	23.5	0.6	0.0005	7.3	0.1	7.7	0.2	7.9	0.2	8.6	0.2	<0.0001
Girls	14.8	0.3	15.4	0.4	15.8	0.3	17.5	0.4	<0.0001	6.9	0.1	7.4	0.2	7.7	0.2	8.3	0.2	<0.0001
**Age**																		
12–14 years	17.1	0.3	18.0	0.4	18.2	0.4	19.8	0.5	<0.0001	7.0	0.1	7.5	0.2	7.8	0.2	8.2	0.2	<0.0001
15–18 years	18.2	0.4	18.8	0.5	18.6	0.4	21.1	0.6	<0.0001	7.2	0.1	7.6	0.2	7.7	0.1	8.6	0.2	<0.0001
**Region**																		
Urban	17.9	0.3	18.4	0.3	18.4	0.3	20.7	0.4	<0.0001	7.2	0.1	7.6	0.1	7.8	0.1	8.5	0.2	<0.0001
Rural	16.9	0.5	18.1	0.7	18.3	0.5	20.2	1.1	0.0051	7.0	0.2	7.6	0.3	8.0	0.2	8.2	0.4	0.0010
**Household income**																		
Lowest	16.3	1.0	17.7	0.7	16.7	0.8	17.6	0.8	0.5505	7.0	0.3	7.7	0.3	7.4	0.3	7.6	0.3	0.2303
Medium-low	17.9	0.5	17.6	0.6	17.1	0.5	20.1	0.9	0.0834	7.4	0.2	7.4	0.2	7.6	0.2	8.5	0.3	0.0032
Medium-high	17.8	0.5	18.5	0.6	19.2	0.5	21.3	0.6	<0.0001	7.1	0.2	7.6	0.2	8.0	0.2	8.6	0.2	<0.0001
Highest	18.5	0.5	19.5	0.7	19.8	0.6	21.7	0.8	<0.0001	7.3	0.2	7.9	0.2	8.2	0.2	8.8	0.3	<0.0001

SE, standard error. ^1^ The statistical analysis accounted for the complex sampling design effect and used appropriate sample weights. ^2^ Means (SE) and *p* trends were obtained from the multiple regression after adjustment for gender, age, region, household income, and total energy intake, where applicable. ^3^ Means (SE) and *p* trends were obtained from the multiple regression after adjustment for gender, age, region, and household income, where applicable.

**Table 4:** Trends in monounsaturated fatty acids (MUFA) intake among Korean adolescents during 2007–2017 ^1^.

Characteristics	MUFA (g) ^2^									MUFA (% kcal) ^3^								
	2007–2009		2010–2012		2013–2015		2016–2017		*p*-Trend	2007–2009		2010–2012		2013–2015		2016–2017		*p*-Trend
	Mean	SE	Mean	SE	Mean	SE	Mean	SE		Mean	SE	Mean	SE	Mean	SE	Mean	SE	
**Total**	17.2	0.3	18.0	0.3	18.7	0.3	20.7	0.4	<0.0001	6.8	0.1	7.3	0.1	7.9	0.1	8.4	0.2	<0.0001
**Gender**																		
Boys	19.6	0.4	20.5	0.5	21.3	0.4	23.6	0.7	<0.0001	6.9	0.1	7.4	0.2	8.0	0.2	8.6	0.2	<0.0001
Girls	14.4	0.3	15.3	0.4	15.9	0.3	17.4	0.4	<0.0001	6.7	0.1	7.3	0.2	7.7	0.2	8.2	0.2	<0.0001
**Age**																		
12–14 years	16.4	0.3	17.1	0.4	18.2	0.4	19.8	0.5	<0.0001	6.6	0.1	7.0	0.1	7.8	0.2	8.1	0.2	<0.0001
15–18 years	17.7	0.4	18.8	0.5	19.3	0.4	21.4	0.6	<0.0001	6.9	0.1	7.6	0.2	8.0	0.1	8.6	0.2	<0.0001
**Region**																		
Urban	17.3	0.3	18.0	0.3	18.8	0.3	20.7	0.5	<0.0001	6.9	0.1	7.4	0.1	7.9	0.1	8.4	0.2	<0.0001
Rural	16.3	0.4	17.8	0.7	18.5	0.5	20.7	1.3	<0.0001	6.6	0.2	7.4	0.3	8.0	0.2	8.4	0.4	<0.0001
**Household income**																		
Lowest	16.5	0.9	17.4	0.8	17.3	0.7	19.0	0.8	0.0697	6.9	0.3	7.6	0.3	7.6	0.3	8.2	0.3	0.0059
Medium-low	17.2	0.4	17.4	0.6	17.1	0.5	20.2	1.0	0.0124	7.0	0.2	7.2	0.2	7.5	0.2	8.4	0.4	0.0001
Medium-high	17.0	0.4	18.3	0.6	19.4	0.5	21.5	0.7	<0.0001	6.6	0.2	7.2	0.2	8.0	0.2	8.6	0.3	<0.0001
Highest	17.7	0.5	18.7	0.7	20.3	0.6	21.1	0.8	<0.0001	6.9	0.2	7.5	0.3	8.3	0.2	8.4	0.3	<0.0001

SE, standard error. ^1^ The statistical analysis accounted for the complex sampling design effect and used appropriate sample weights. ^2^ Means (SE) and *p* trends were obtained from the multiple regression after adjustment for gender, age, region, household income, and total energy intake, where applicable. ^3^ Means (SE) and *p* trends were obtained from the multiple regression after adjustment for gender, age, region, and household income, where applicable.

**Table 5:** Trends in polyunsaturated fatty acids (PUFA) intake among Korean adolescents during 2007–2017 ^1^.

Characteristics	2007–2009		2010–2012		2013–2015		2016–2017		*p*-Trend	2007–2009		2010–2012		2013–2015		2016–2017		*p*-Trend
	Mean	SE	Mean	SE	Mean	SE	Mean	SE		Mean	SE	Mean	SE	Mean	SE	Mean	SE	
	**PUFA (g) ^2^**									**PUFA (% kcal) ^3^**								
**Total**	12.4	0.2	12.1	0.2	12.5	0.2	13.2	0.3	0.0050	5.0	0.1	5.0	0.1	5.3	0.1	5.4	0.1	0.0003
**Gender**																		
Boys	13.7	0.3	13.3	0.3	13.9	0.3	14.6	0.4	0.0250	5.0	0.1	4.9	0.1	5.3	0.1	5.4	0.1	0.0019
Girls	11.0	0.3	10.7	0.3	10.9	0.3	11.7	0.4	0.0812	5.1	0.1	5.0	0.1	5.3	0.1	5.4	0.2	0.0371
**Age**																		
12–14 years	11.5	0.2	11.4	0.3	12.1	0.3	13.0	0.4	<0.0001	4.7	0.1	4.7	0.1	5.1	0.1	5.4	0.1	<0.0001
15–18 years	13.2	0.3	12.7	0.3	13.0	0.3	13.6	0.4	0.2610	5.4	0.1	5.2	0.1	5.4	0.1	5.5	0.1	0.1584
**Region**																		
Urban	12.4	0.2	12.0	0.2	12.5	0.2	13.1	0.3	0.0222	5.1	0.1	4.9	0.1	5.3	0.1	5.4	0.1	0.0024
Rural	12.2	0.4	12.0	0.4	12.1	0.5	13.9	0.7	0.0711	5.0	0.2	5.1	0.2	5.2	0.2	5.7	0.3	0.0420
**Household income**																		
Lowest	11.9	0.5	11.5	0.5	12.0	0.6	13.7	0.9	0.0865	5.3	0.3	4.9	0.2	5.4	0.3	5.9	0.4	0.1251
Medium-low	12.5	0.4	12.0	0.4	11.8	0.4	12.5	0.5	0.7853	5.2	0.2	5.1	0.2	5.1	0.2	5.2	0.2	0.8937
Medium-high	12.1	0.3	12.3	0.4	12.6	0.4	13.6	0.5	0.0042	4.8	0.1	4.9	0.2	5.2	0.1	5.5	0.2	0.0002
Highest	12.7	0.3	12.0	0.4	13.0	0.4	13.1	0.4	0.2268	5.0	0.1	4.9	0.1	5.4	0.1	5.2	0.2	0.0888
	**n-3 FA (g) ^2^**									**n-3 FA (% kcal) ^3^**								
**Total**	1.52	0.03	1.44	0.04	1.46	0.03	1.60	0.05	0.2437	0.63	0.01	0.60	0.02	0.62	0.01	0.66	0.02	0.1266
**Gender**																		
Boys	1.65	0.05	1.54	0.05	1.60	0.05	1.75	0.07	0.2237	0.61	0.02	0.58	0.02	0.61	0.02	0.64	0.02	0.2232
Girls	1.38	0.05	1.32	0.06	1.31	0.05	1.44	0.06	0.6297	0.64	0.02	0.62	0.02	0.63	0.02	0.68	0.03	0.2973
**Age**																		
12–14 years	1.46	0.04	1.37	0.05	1.44	0.04	1.67	0.08	0.0222	0.60	0.02	0.57	0.02	0.61	0.02	0.70	0.04	0.0062
15–18 years	1.57	0.05	1.49	0.06	1.48	0.05	1.57	0.06	0.8264	0.65	0.02	0.62	0.02	0.63	0.02	0.64	0.02	0.9127
**Region**																		
Urban	1.52	0.03	1.43	0.04	1.48	0.03	1.60	0.05	0.2207	0.63	0.02	0.60	0.02	0.63	0.01	0.66	0.02	0.1097
Rural	1.52	0.07	1.42	0.09	1.36	0.06	1.61	0.11	0.9486	0.64	0.03	0.61	0.04	0.58	0.03	0.69	0.05	0.9700
**Household income**																		
Lowest	1.39	0.08	1.32	0.09	1.37	0.09	1.66	0.17	0.1697	0.62	0.04	0.57	0.04	0.63	0.05	0.70	0.06	0.2115
Medium-low	1.59	0.06	1.44	0.08	1.38	0.06	1.55	0.08	0.3745	0.65	0.03	0.62	0.03	0.60	0.02	0.65	0.03	0.5617
Medium-high	1.50	0.06	1.53	0.08	1.44	0.05	1.67	0.08	0.2028	0.62	0.02	0.62	0.03	0.61	0.02	0.69	0.03	0.1044
Highest	1.54	0.06	1.38	0.07	1.56	0.07	1.53	0.08	0.5712	0.62	0.03	0.58	0.03	0.65	0.03	0.63	0.04	0.4566
	**n-6 FA (g) ^2^**									**n-6 FA (% kcal) ^3^**								
**Total**	11.0	0.2	10.8	0.2	11.1	0.2	11.6	0.2	0.0092	4.4	0.1	4.4	0.1	4.7	0.1	4.7	0.1	0.0006
**Gender**																		
Boys	12.2	0.3	11.9	0.3	12.4	0.3	12.9	0.4	0.0442	4.4	0.1	4.4	0.1	4.7	0.1	4.7	0.1	0.0030
Girls	9.7	0.2	9.5	0.2	9.7	0.3	10.2	0.3	0.0873	4.5	0.1	4.4	0.1	4.7	0.1	4.7	0.1	0.0491
**Age**																		
12–14 years	10.2	0.2	10.2	0.2	10.7	0.2	11.3	0.3	0.0001	4.1	0.1	4.2	0.1	4.5	0.1	4.6	0.1	<0.0001
15–18 years	11.7	0.3	11.3	0.3	11.6	0.3	12.1	0.3	0.2635	4.7	0.1	4.6	0.1	4.8	0.1	4.9	0.1	0.1616
**Region**																		
Urban	11.0	0.2	10.7	0.2	11.1	0.2	11.5	0.2	0.0460	4.5	0.1	4.4	0.1	4.7	0.1	4.7	0.1	0.0054
Rural	10.8	0.3	10.7	0.4	10.9	0.4	12.3	0.6	0.0433	4.4	0.1	4.5	0.2	4.7	0.2	5.0	0.3	0.0347
**Household income**																		
Lowest	10.6	0.5	10.3	0.5	10.7	0.5	12.0	0.8	0.1054	4.7	0.2	4.4	0.2	4.8	0.2	5.2	0.3	0.1182
Medium-low	11.1	0.3	10.6	0.4	10.5	0.3	11.0	0.4	0.7154	4.6	0.1	4.5	0.1	4.6	0.1	4.6	0.2	0.9915
Medium-high	10.7	0.3	10.9	0.4	11.3	0.3	11.9	0.4	0.0045	4.2	0.1	4.4	0.1	4.7	0.1	4.8	0.2	0.0002
Highest	11.3	0.3	10.8	0.3	11.5	0.3	11.5	0.4	0.3343	4.4	0.1	4.4	0.1	4.8	0.1	4.6	0.1	0.1534

n-3 FA, n-3 fatty acids; n-6 FA, n-6 fatty acids; SE, standard error. ^1^ The statistical analysis accounted for the complex sampling design effect and used appropriate sample weights. ^2^ Means (SE) and *p* trends were obtained from the multiple regression after adjustment for gender, age, region, household income, and total energy intake, where applicable. ^3^ Means (SE) and *p* trends were obtained from the multiple regression after adjustment for gender, age, region, and household income, where applicable.

## References

[1] GBD 2017 Causes of Death Collaborators (2018). Global, regional, and national age-sex-specific mortality for 282 causes of death in 195 countries and territories, 1980–2017: A systematic analysis for the Global Burden of Disease Study 2017. Lancet.

[2] Eckel R.H., Jakicic J.M., Ard J.D., de Jesus J.M., Houston Miller N., Hubbard V.S., Lee I.M., Lichtenstein A.H., Loria C.M., Millen B.E. (2014). 2013 AHA/ACC guideline on lifestyle management to reduce cardiovascular risk: A report of the American College of Cardiology/American Heart Association Task Force on Practice Guidelines. Circulation.

[3] Lichtenstein A.H., Appel L.J., Brands M., Carnethon M., Daniels S., Franch H.A., Franklin B., Kris-Etherton P., Harris W.S., Howard B. (2006). Diet and lifestyle recommendations revision 2006: A scientific statement from the American Heart Association Nutrition Committee. Circulation.

[4] U.S. Department of Health and Human Services and U.S. Department of Agriculture (2015). 2015–2020 Dietary Guidelines for Americans.

[5] Food and Agriculture Organization (2010). Fats and Fatty Acids in Human Nutrition. Report of an Expert Consultation.

[6] Kubena K.S. (2011). Metabolic syndrome in adolescents: Issues and opportunities. J. Am. Diet Assoc..

[7] May A.L., Kuklina E.V., Yoon P.W. (2012). Prevalence of cardiovascular disease risk factors among US adolescents, 1999–2008. Pediatrics.

[8] Liang Y., Hou D., Zhao X., Wang L., Hu Y., Liu J., Cheng H., Yang P., Shan X., Yan Y. (2015). Childhood obesity affects adult metabolic syndrome and diabetes. Endocrine.

[9] Saarikoski L.A., Juonala M., Huupponen R., Viikari J.S.A., Lehtimäki T., Jokinen E., Hutri-Kähönen N., Taittonen L., Laitinen T., Raitakari O.T. (2017). Low serum adiponectin levels in childhood and adolescence predict increased intima-media thickness in adulthood. The Cardiovascular Risk in Young Finns Study. Ann. Med..

[10] Baek Y., Hwang J.Y., Kim K., Moon H.K., Kweon S., Yang J., Oh K., Shim J.E. (2015). Dietary intake of fats and fatty acids in the Korean population: Korea National Health and Nutrition Examination Survey, 2013. Nutr. Res. Pr..

[11] Cui Z., Dibley M.J. (2012). Trends in dietary energy, fat, carbohydrate and protein intake in Chinese children and adolescents from 1991 to 2009. Br. J. Nutr..

[12] Rippin H.L., Hutchinson J., Jewell J., Breda J.J., Cade J.E. (2019). Child and adolescent nutrient intakes from current national dietary surveys of European populations. Nutr. Res. Rev..

[13] U.S. Department of Agricultur, Agricultural Research Service (2018). Energy Intakes: Percentages of Energy from Protein, Carbohydrate, Fat, and Alcohol, by Gender and Age, What We Eat in America, NHANES 2015–2016.

[14] Abdul Majid H., Ramli L., Ying S.P., Su T.T., Jalaludin M.Y., Abdul Mohsein N.A.-S. (2016). Dietary intake among adolescents in a middle-income country: An outcome from the Malaysian Health and Adolescents Longitudinal Research Team Study (the MyHeARTs Study). PLoS ONE.

[15] Pot G.K., Prynne C.J., Roberts C., Olson A., Nicholson S.K., Whitton C., Teucher B., Bates B., Henderson H., Pigott S. (2012). National Diet and Nutrition Survey: Fat and fatty acid intake from the first year of the rolling programme and comparison with previous surveys. Br. J. Nutr..

[16] Ervin R.B., Ogden C.L. (2013). Trends in intake of energy and macronutrients in children and adolescents from 1999–2000 through 2009–2010. Nchs. Data Brief..

[17] Charzewska J., Chwojnowska Z., Wajszczyk B., Chabros E. (2015). Twenty four year time trends in fats and cholesterol intake by adolescents. Warsaw Adolescents Study. Anthropol. Rev..

[18] Libuda L., Alexy U., Kersting M. (2014). Time trends in dietary fat intake in a sample of German children and adolescents between 2000 and 2010: Not quantity, but quality is the issue. Br. J. Nutr..

[19] Meyer B.J. (2016). Australians are not meeting the recommended intakes for omega-3 long chain polyunsaturated fatty acids: Results of an analysis from the 2011–2012 National Nutrition and Physical Activity Survey. Nutrients.

[20] Ley S.H., Hamdy O., Mohan V., Hu F.B. (2014). Prevention and management of type 2 diabetes: Dietary components and nutritional strategies. Lancet.

[21] Korea Centers for Disease Control and Prevention (2018). Korea Health Statistics 2017: Korea National Health and Nutrition Examination Survey (KNHANES Ⅶ-2).

[22] Song S., Shim J.E., Song W.O. (2019). Trends in total fat and fatty acid intakes and chronic health conditions in Korean adults over 2007–2015. Public Health Nutr..

[23] Song S., Shim J.E. (2019). Evaluation of total fat and fatty acids intakes in the Korean adult population using data from the 2016–2017 Korea National Health and Nutrition Examination Surveys. Korean J. Community Nutr..

[24] Kwon E., Nah E.-H. (2016). Secular trends in height, weight and obesity among Korean children and adolescents in 2006–2015. Korean J. Health Educ. Promot..

[25] Kweon S., Kim Y., Jang M.J., Kim Y., Kim K., Choi S., Chun C., Khang Y.H., Oh K. (2014). Data resource profile: The Korea National Health and Nutrition Examination Survey (KNHANES). Int. J. Epidemiol..

[26] National Institute of Agricultural Sciences (2006). Food Composition Table.

[27] National Institute of Agricultural Sciences (2011). Food Composition Table.

[28] National Institute of Agricultural Sciences (2016). Food Composition Table.

[29] Yoon M.O., Kim K., Hwang J.-Y., Lee H.S., Son T.Y., Moon H.-K., Shim J.E. (2014). Development of a fatty acids database using the Korea National Health and Nutrition Examination Survey data. J. Nutr. Health.

[30] The Korean Nutrition Society (2016). Dietary Reference Intakes for Koreans 2015.

[31] Saito A., Imai S., Htun N.C., Okada E., Yoshita K., Yoshiike N., Takimoto H. (2018). The trends in total energy, macronutrients and sodium intake among Japanese: Findings from the 1995–2016 National Health and Nutrition Survey. Br. J. Nutr..

[32] Shen X., Fang A., He J., Liu Z., Guo M., Gao R., Li K. (2017). Trends in dietary fat and fatty acid intakes and related food sources among Chinese adults: A longitudinal study from the China Health and Nutrition Survey (1997–2011). Public Health Nutr..

[33] Harika R.K., Cosgrove M.C., Osendarp S.J.M., Verhoef P., Zock P.L. (2011). Fatty acid intakes of children and adolescents are not in line with the dietary intake recommendations for future cardiovascular health: A systematic review of dietary intake data from thirty countries. Br. J. Nutr..

[34] Calvo-Lerma J., Hulst J., Boon M., Martins T., Ruperto M., Colombo C., Fornés-Ferrer V., Woodcock S., Claes I., Asseiceira I. (2019). The relative contribution of food groups to macronutrient intake in children with cystic fibrosis: A European multicenter assessment. J. Acad. Nutr. Diet.

[35] Aranceta J., Perez-Rodrigo C. (2012). Recommended dietary reference intakes, nutritional goals and dietary guidelines for fat and fatty acids: A systematic review. Br. J. Nutr..

